# A Nosocomial Respiratory Infection Outbreak of Carbapenem-Resistant *Escherichia coli* ST131 With Multiple Transmissible *bla*_*KPC–*2_ Carrying Plasmids

**DOI:** 10.3389/fmicb.2020.02068

**Published:** 2020-09-11

**Authors:** Lin Gong, Na Tang, Dongke Chen, Kaiwen Sun, Ruiting Lan, Wen Zhang, Haijian Zhou, Min Yuan, Xia Chen, Xiaofei Zhao, Jie Che, Xuemei Bai, Yunfei Zhang, Hongtao Xu, Timothy R. Walsh, Jinxing Lu, Jianguo Xu, Juan Li, Jie Feng

**Affiliations:** ^1^State Key Laboratory for Infectious Disease Prevention and Control, Collaborative Innovation Center for Diagnosis and Treatment of Infectious Disease, National Institute for Communicable Disease Control and Prevention, China CDC, Beijing, China; ^2^Wuhan Centers for Disease Prevention and Control, Wuhan, China; ^3^State Key Laboratory of Microbial Resources, Institute of Microbiology, Chinese Academy of Sciences, Beijing, China; ^4^College of Life Science, University of Chinese Academy of Sciences, Beijing, China; ^5^Department of Laboratory Medicine, Beijing Hospital, National Center of Gerontology, Beijing, China; ^6^School of Biotechnology and Biomolecular Sciences, University of New South Wales, Sydney, NSW, Australia; ^7^Department of Medical, Microbiology, School of Medicine, Cardiff University, Cardiff, United Kingdom

**Keywords:** *bla*_*KPC–*2_, *Escherichia coli* ST131, outbreak, respiratory infection, transmissible plasmids

## Abstract

*Escherichia coli* sequence type 131 (ST131) is well known for its multidrug resistance profile. Carbapenems have been considered the treatment of choice for *E. coli* ST131 infections, and resistance to carbapenems is emerging due to the acquisition of carbapenemase-encoding genes. In this study, 45 carbapenem-resistant *E. coli* strains were collected in a hospital. The resistance mechanisms, plasmid profiles, and genetic relatedness of these strains were determined. Phylogenetic relationships between these strains were assessed by molecular profiling and aligned with patient clinical details. The genetic context of *bla*_*KPC–*2_ was analyzed to trace the potential dissemination of *bla*_*KPC–*2_. The 45 carbapenem-resistant *E. coli* ST131 strains were closely related. Initially prevalent only in a single ward, ST131 subsequently spread to other ward, resulting in a respiratory infection outbreak of carbapenem-resistant *E. coli* ST131. Eight of the 30 patients died within 28 days of the first isolation of *E. coli* ST131. The *bla*_*KPC–*2_-positive plasmid profiles suggest that the carbapenem resistance was due to the acquisition by *E. coli* ST131 of transmissible plasmids pE0272_KPC and pE0171_KPC carrying *bla*_*KPC–*2_. Additionally, diverse multidrug resistance elements were transferred and rearranged between these plasmids mediated by IS26. Our research indicates that clinical attention should be paid to the importance of *E. coli* ST131 in respiratory infections and the spread of *bla*_*KPC*_-carrying *E. coli* ST131.

## Introduction

*Escherichia coli* sequence type 131 (ST131) is one of the most widespread and successful extra-intestinal pathogenic *E. coli* (ExPEC) clones that can cause community- and hospital-acquired urinary tract infections (UTIs), abdominal and pelvic infections, and bacteremia with high morbidity and mortality (Nicolas-Chanoine et al., 2014). *E. coli* ST131 has developed quinolone resistance associated with mutations within the *gyrA* and *parC* genes, as well as resistance against extended-spectrum β-lactam antibiotics, mainly due to the acquisition of CTX-M-15 (Price et al., 2013; Petty et al., 2014; Ben Zakour et al., 2016). Thus, *E. coli* ST131 has raised public health concerns, due not only to its high pathogenicity potential, but also to its increasing antimicrobial resistance.

Currently, carbapenems have been considered the treatment of choice for *E. coli* ST131 infections. Although carbapenem resistance is rarely reported (Xu et al., 2015; Zhang et al., 2018), the rate at which bacteria are becoming resistant to carbapenems is slowly growing, due mainly to the acquisition of New Delhi metallo-β-lactamase (NDM) or oxacillinase (OXA)-48-like β-lactamases and *Klebsiella pneumoniae* carbapenemase (KPC) (Perry et al., 2011; Li et al., 2014; Peirano et al., 2014; Chen et al., 2016; Zhang et al., 2018). KPC is the most prevalent carbapenemase in *K. pneumoniae*, and *bla*_*KPC*_ is typically found within the transposable elements of transferable plasmids in *K. pneumoniae*. Tn4401 is a Tn3-based transposon that carries the *bla*_*KPC*_ gene (Naas et al., 2008). Tn1722 has also been found carrying *bla*_*KPC*_ in *K. pneumonia* strains from China, which partially harbors the structure of Tn4401 (Shen et al., 2009). Thus far, there are only sporadic reports on the detection of *bla*_*KPC*_ in *E. coli* ST131, and these few reports are unrelated to outbreaks (Peirano et al., 2014; Baraniak et al., 2015; Piazza et al., 2016).

Extraintestinal infections of *E. coli* occur mainly in the urinary tract. To date, there have been few reports of *E. coli* ST131 causing respiratory infections. A recent study reported the emergence of CTX-M-15-producing *E. coli* ST131, which caused hospital-acquired pneumonia in regions of Asia (Cha et al., 2017). The virulence factors of ExPEC are broad and associated with site-specific infections. Invasion of brain endothelium proteins, outer membrane protein A, the bacterial capsule *fimH*, and cytotoxic necrotizing factor 1 (*cnf1*) were associated with invasion of the blood-brain barrier (Croxen and Finlay, 2010). Salmochelin receptor *iroN*, adhesin factors *sfa*/*foc* and *papGIII*, and toxin factors *hlyC* and *cnf*1 were found to be significantly over-represented in pneumonia *E. coli* strains, compared with infectious strains found in other sites (La Combe et al., 2019).

Here, we report on a molecular epidemiological study of a nosocomial outbreak of respiratory infections caused predominantly by carbapenem-resistant *E. coli* ST131 in a Chinese hospital during 2012 and 2013. We demonstrate that the underlying resistance was due to the acquisition by *E. coli* ST131 of multiple transmissible plasmids carrying *bla*_*KPC–*2_.

## Materials and Methods

### Ethics Statement

The human specimens were acquired with consent from the patients. This study was reviewed and approved by the ethics committee of the National Institute for Communicable Disease Control and Prevention, China CDC, according to the medical research regulations of the Ministry of Health, China. This research was conducted within China.

### Strain Collection

On July 1, 2012, one strain of *E. coli* was isolated from a sputum sample from a pneumonia patient presenting with expiratory dyspnea in the geriatrics department at a hospital in Beijing. This strain showed resistance to carbapenems. Since then, carbapenem-resistant *E. coli* strains were frequently isolated in four wards of the geriatric and neurology departments until December 28, 2013. A total of 45 strains were included in the study. Some of the *E. coli* strains were collected from the same patient on different days. The bacterial strains were cultured on Luria-Bertani (LB) (Oxoid, Wesel, Germany) plates at 37°C for 24 h.

### MIC Determination and Carbapenem-Resistance Gene Screening

To determine the susceptibility of 45 strains to antibiotics, minimum inhibitory concentrations (MICs) were determined by broth microdilution, according to the protocol of the Clinical and Laboratory Standards Institute^1^.

To unveil the carbapenem-resistant mechanism, carbapenem resistance genes (*bla*_*OXA–*48_, *bla*_*KPC*_, *bla*_*VIM*_, *bla*_*IMP*_, and *bla*_*NDM*_) were screened, and the primers used for the PCR reactions are listed in Supplementary Table S1 (Poirel et al., 2011). Genomic DNA used as PCR template DNA was extracted from culture grown overnight on LB broth using the TIANamp Bacteria DNA Kit (TIANGEN, Beijing, China). Amplification was carried out with the following thermal cycling regimen: 3 min at 95°C, followed by 35 cycles of amplification consisting of 15 s at 95°C, 15 s at 52°C, and 60 s at 72°C, with 5 min at 72°C for the final extension. Amplicons were visualized after electrophoresis at 110 V for 30 min on a 0.7% agarose gel. All positive PCR products were sequenced using an ABI3730 sequencer (Applied Biosystems, Foster City, CA, United States), and their sequences were analyzed using the National Center for Biotechnology Information (NCBI) BLAST program.

### *Xba*I-PFGE, S1-PFGE, and Southern Blot Hybridization

For strain typing, whole-cell *E. coli* DNA embedded in agarose gel plugs was digested with XbaI (Takara Bio Inc.) and was separated by Pulse-field gel electrophoresis (PFGE) using a CHEF-DR III apparatus (Bio-Rad, Hercules, CA, United States), as previously described (Beutin et al., 2005). Plasmids were analyzed and sized using the S1-PFGE nuclease method. Large fragments from the restriction enzyme digest were separated by PFGE using a CHEF-DR III apparatus (Bio-Rad) for 7 h at 6 V/cm and 14°C, with initial and final pulse times of 5 and 20 s, respectively. Southern hybridization was performed with the *bla*_*KPC–*2_ gene as a probe to ascertain the *bla*_*KPC–*2_-positive plasmid, using the electrochemiluminescence direct nucleic acid labeling and detection system per the manufacture’s operation protocol (GE Healthcare, Buckinghamshire, United Kingdom).

### Genome Sequencing and Annotation

Genomic DNA was extracted using the Wizard Genomic DNA Purification Kit (Promega, United States) for genome sequencing. DNA paired-end sequencing was performed on an Illumina HiSeq 2,000 (Illumina, San Diego, CA, United States) with a read length of 2 × 100 bp to obtain the draft genomes of 45 strains. The *de novo* genome was assembled from the Illumina data using Velvet ver. 1.2.10 (Zerbino and Birney, 2008). A Pacific Biosciences RSII DNA sequencing system (PacBio, Menlo Park, CA, United States) was used as the sequencing platform to sequence complete genomes of strains E0171, E0272, and E02162, which are representative of the main types of *bla*_*KPC–*2_-positive plasmids in the 45 strains, according to S1-PFGE southern blot profiles. *De novo* assembly of the Pacific Biosciences long reads was performed using the Hierarchical Genome Assembly Process (HGAP_Assembly.2) algorithm in the single molecule real-time (SMRT) Portal, with default parameters.

The assembled genome and plasmids were annotated using Prokka 1.12.21 (Seemann, 2014). The sequence type of the strains was predicted using BLAST with the multi-locus sequence typing (MLST) database^2^. The plasmid incompatibility groups (Inc.) were assigned using the PlasmidFinder Web tool (Carattoli et al., 2014). Antibiotic resistance genes were annotated using Resistance Gene Identifier (RGI) ver. 3.2.0 (Jia et al., 2017). The ISfinder database and Integron Database were used to annotate mobile elements (Siguier et al., 2006; Moura et al., 2009). The schematic map of linear comparison and circular diagram of sequenced plasmids were drawn using in-house Perl scripts and Inkscape version 0.92^3^. The sequenced plasmids was compared using BLAST with nucleotide identity >90% and BLAST Ring Image Generator (BRIG) ver 0.95 (Alikhan et al., 2011). Sequence reads were mapped onto the backbone of plasmid pE0272_KPC, pE0171_KPC, and pE02162_KPC using Bowtie2 to determine the presence of plasmids in 45 strains (Langmead and Salzberg, 2012), and the mapped region on the plasmid was visualized using Artemis (Carver et al., 2012). The genes associated with virulence were determined by BLAST with a cutoff of nucleotide identity ≥95% and coverage ≥95% against the 38 ExPEC virulence genes (Dale and Woodford, 2015) (Supplementary Table S2). Fisher’s exact test was used to compare the distribution of virulence genes in strains isolated from sputum and urine samples. We considered *p* < 0.05 to be significant.

### Phylogenetic Analysis

The kSNP alignment-free method was used to generate an independent phylogenetic tree based on single-nucleotide polymorphism (SNP) to interpret the phylogenetic relationships of the ST131 strains (Gardner et al., 2015). The draft genomes of 45 strains, together with 197 strains of *E. coli* ST131 downloaded from the NCBI database (Supplementary Table S3. The genomes from Sequence Read Archive database were assembled using Velvet ver. 1.2.10), were used as the input to kSNP version 3.0 with default parameters and a *k*-mer value of 19, as predicted by the kSNP-associated Kchooser script, to generate an independent phylogenetic tree based on SNPs (Gardner et al., 2015). The *fimH*, *parC*, and *gyrA* allelic profiles were obtained by BlastX according to the classification standard of Johnson et al. (2013).

A whole-genome, SNP-based phylogenetic analysis was performed to infer the phylogenetic relationships of the 45 strains. Strain FC10268 was selected as a reference genome; this strain was tightly clustered with the 45 strains in a phylogenetic tree of 242 ST131 *E. coli* strains. Bowtie2 was used to map the Illumina reads of the 45 strains to the genome of strain FC10268 and to generate BAM files for SNP calling using FreeBayes with a min-alternate-fraction of 0.2 (Garrison and Marth, 2012; Langmead and Salzberg, 2012). High-quality SNPs were filtered according to the following criteria: (1) quality scores >20; (2) >30 reads covering the SNP site; and (3) >70% of the reads support the SNP. SNPs from a manually curated plasmid contig using nucleotide BLAST were considered unreliable and were excluded from further analyses. MEGA6 software was used to infer the phylogenetic tree of 136 high-quality SNPs using the maximum parsimony (MP) method (Tamura et al., 2013). The MP tree was obtained using the Tree-Bisection-Regrafting algorithm with search level 1 (Nei and Kumar, 2000), in which the initial trees were obtained by random addition of sequences (ten replicates). The tree was drawn to scale, with branch lengths calculated using the average pathway method (Nei and Kumar, 2000), in which the dimensional units were the number of changes over the entire sequence. All trees were viewed using Interactive Tree of Life (iTOL) (Letunic and Bork, 2016).

### Plasmid Conjugal Transfer Experiment

To verify the mobility of the *bla*_*KPC*–2_-carrying plasmid, plasmid conjugal transfer experiments were carried out using rifampicin-resistant *E. coli* EC600 as the recipient, and strains E0272 and E0171 as the donors. Overnight cultures of the donor strain (600 μL) and recipient strain (400 μL) were mixed and collected by centrifugation (2,500 rcf, 10 min). The mixture was incubated on LB agar plates for 24 h at 37°C. LB agar plates containing rifampicin (100 μg/mL) plus meropenem (2 μg/mL) were used for the selection of carbapenemase-positive *E. coli* transconjugants. To determine the presence of the plasmid in transconjugants, primers were designed based on the three backbone genes of each plasmid (Supplementary Table S4) and amplified by PCR as described above. The PCR products were detected using agarose gel electrophoresis.

### Data Availability

The Illumina sequence reads from all strains were deposited in the Sequence Read Archive database under accession numbers PRJNA514844. The complete nucleotide sequences of pE0171_KPC, pE0272_2, pE0272_KPC, and pE02162_KPC were submitted to GenBank under accession numbers MK370988, MK370989, MK370990, and MK370991, respectively.

## Results

### Nosocomial Outbreak of Carbapenem-Resistant *E. coli* Infections in a Geriatric Department

**FIGURE 1:**
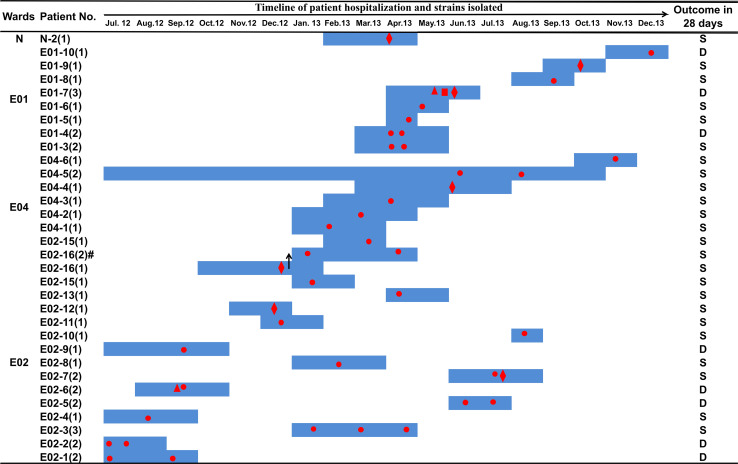
Clinical features of patients from whom ST131 *E. coli* carrying *bla*_*KPC–*2_ was isolated. Dark blue boxes indicate the period of hospitalization. Red dots indicate the time of *bla*_*KPC–*2_-positive *E. coli* isolation. The shapes of the red dots indicate the type of samples: circle, sputum or nasopharyngeal swab sample; diamond, urine; triangle, blood; square, drainage. The black arrows indicate a change of ward: patient number E02-16 was transferred from ward E02 to ward E04 on December 27, 2012. Abbreviations: D, death; S, survival.

From July 2012 to December 2013, a total of 45 carbapenem-resistant *E. coli* strains were collected from 30 patients. The 45 strains were all isolated from infections, 34 of which (75.6%) were from sputum or nasopharyngeal samples, eight (17.8%) were from urine samples, two (4.4%) were isolated from blood cultures, and one (2.2%) isolate was from an ascitic fluid sample of a patient presenting with sepsis (Figure 1 and Supplementary Table S5). Of these 30 patients, 27 had been administered biapenem at 0.6 g per day for 3–30 days. Eight patients died within 28 days of the first isolation of carbapenem-resistant *E. coli* (Supplementary Table S5). All 45 strains exhibited medium to high MICs of carbapenem (Supplementary Table S5). All (100%) were resistant to ertapenem, 35 (77.8%) were resistant to imipenem, and 25 (51.1%) were resistant to meropenem (Supplementary Table S5). Furthermore, these strains exhibited resistance to most other classes of antibiotics, such as non-carbapenem β-lactams, quinolones, aminoglycosides, and tetracyclines, but were sensitive to chloramphenicol (Supplementary Table S5).

All *E. coli* strains carried the *bla*_*KPC–*2_ gene, while other carbapenem resistance genes were not detected (Supplementary Table S5), and all strains shared highly similar *Xba*I-PFGE patterns (Supplementary Figure S1A), suggesting that they may belong to the same clone. S1-PFGE southern blot hybridization demonstrated that *bla*_*KPC–*2_ was located on plasmids of various sizes, ranging from 130 to 270 kb (Supplementary Figures S1B,C). Most strains carried a single *bla*_*KPC–*2_-positive plasmid, although strain E02-11 contained two *bla*_*KPC–*2_-positive plasmids. According to the sizes of the *bla*_*KPC–*2_-positive plasmids, there were two main plasmid types in 45 strains: a 147-kb plasmid, which was the predominant *bla*_*KPC–*2_-positive plasmid (56%, 25/45), and a 130-kb plasmid (13%, 6/45). Strain E02162 carried the largest *bla*_*KPC–*2_-positive plasmid.

### Genomic Characterization of the Outbreak Strains

We sequenced the 45 strains on an Illumina HiSeq 2000; details of the Illumina reads are provided in Supplementary Table S6. *In silico* MLST analysis showed that all 45 strains belonged to group ST131. We generated a phylogenetic tree based on SNPs using a draft genome of the 45 strains along with 197 *E. coli* ST131 strains from the NCBI database. The 242 total ST131 strains were divided into clades A–C (Supplementary Figure S2). The 45 strains collected from the patients clustered to the C clade, characterized by a *fimH30* allele and the fluoroquinolone resistance alleles *gyrA1AB* and *parC1aAB* (Ben Zakour et al., 2016). We confirmed that our strains also harbored the *fimH30* allele and *gyrA1AB*, and *parC1aAb*. Clade C was further divided into two subclades: C1, characterized by different *bla*_*CTX–M*_ alleles, and C2, characterized by the *bla*_*CTX–M–*15_ gene (Ben Zakour et al., 2016). The 45 strains were clustered in the C1 subclade, and four strains possessed *bla*_*CTX–M–*3_ (Supplementary Figure S2). Furthermore, our strains were tightly clustered with the human clinical strain FC10268 from Beijing, which we used as a reference genome (Supplementary Figure S2).

**FIGURE 2:**
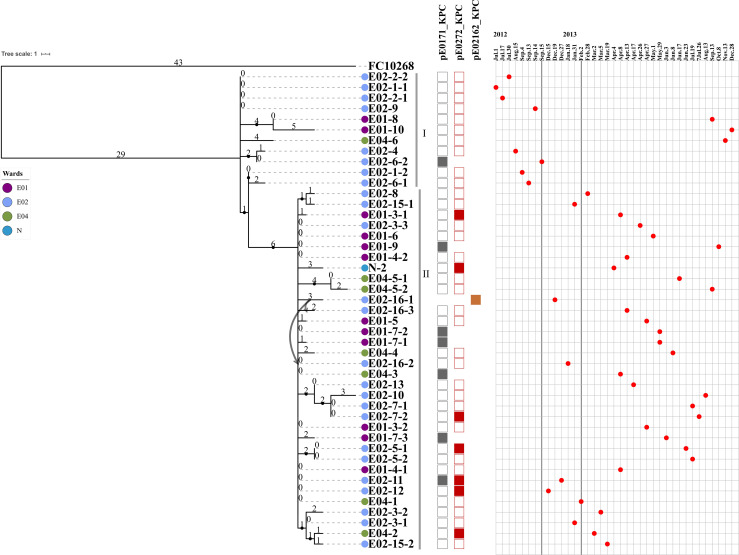
The maximum parsimony phylogenetic tree based on SNP and information about 45 ST131 *E. coli* strains and the reference genome FC10268. Tree number four out of the ten most parsimonious trees (length = 136) is shown. The consistency index, retention index, and composite index were each 1.000000 for all sites and parsimony-informative sites. Only bootstrap (1,000 replicates) values greater than 60% are shown. The strain IDs are labeled using three strings of characters, separated by a hyphen. The first string of characters represents the ward of the patient from whom the strain was isolated, E01, E02, E04, and N1. The second string of characters represents the patient number. The third string of characters represents the strain isolated from that patient. Lavender indicates the E01 ward, light blue indicates the E02 ward, green indicates the E04 ward, and dark blue indicates the N1 ward. The curved lines indicate that patients E02-16 were transferred from the E02 ward to the E04 ward. The SNP number is marked on the middle of branch. The strains of Cluster I and Cluster II are marked with gray vertical lines. Strains carrying the pE0171_KPC plasmid backbone, the pE0272_KPC plasmid backbone, or the pE02162_KPC plasmid backbone are indicated by gray, red, or brown squares, respectively. The *bla*_*KPC–*2_-positive plasmid in the strain is displayed above the column of corresponding color-filled squares. The strains are arranged in the chronological order in which the samples from which they were isolated were collected.

We further analyzed the phylogenetic relationships of the 45 carbapenem-resistant *E. coli* ST131 strains by comparison with the FC10268 reference genome. The strains were clustered into two clusters, separated by six SNPs (Figure 2). The six SNPs were all found in coding regions, including one synonymous and five non-synonymous SNPs (Supplementary Table S7). Cluster I contained eight strains isolated from ward E02, two strains from ward E01, and one from ward E04. All of the strains from ward E02 in Cluster I were isolated before September 15, 2012, indicating that these strains spread within ward E02 only during the early period and were subsequently transmitted to ward E04 and E01.

The first strain of Cluster II, E02-12, was isolated on December 15, 2012, after which the Cluster II strains became dominant in ward E02, suggesting that the Cluster II strains acquired genetic variation that adapted them to external stresses. Historically, patient E02-16, from whom the *bla*_*KPC–*2_-positive strain E02-16-1 was isolated, was transferred from ward E02 to E04 on December 27, 2012. A second *bla*_*KPC–*2_-positive strain E02-16-2 was isolated from patient E02-16 on January 18, 2013, while the patient was resident in ward E04. Then, more *bla*_*KPC–*2_-positive strains were isolated from patients in ward E04. We speculate that the transfer of patient E02-16 led to the spread of Cluster II strains in ward E04. Subsequently, the Cluster II strains were found in four wards, suggesting concomitant transmission across different ward.

The genes associated with virulence were determined by BLAST against the 38 ExPEC virulence genes and nine virulence factors detected in the 45 strains (Supplementary Table S2). The nine genes encode the adhesin siderophore receptor, the *E. coli* common pilus, the type 1 fimbriae, the periplasmic iron binding protein, the yersiniabactin receptor, an outer membrane receptor, β-glucoronidase, a pathogenicity island marker, and D-serine deaminase (Supplementary Tables S2, S8). The distribution of these nine genes in strains from sputum and urine samples did not show significant differences (*p* > 0.05; Supplementary Table S8).

### Genetic Context of Carbapenem Resistance by Plasmid Sequencing

We selected three strains E0171, E0272, and E02162, which were representative for the main types of *bla*_*KPC–*2_-positive plasmids in the 45 strains, according to the S1-PFGE southern blot profiles, to be sequenced using the Pacific Biosciences RSII DNA sequencing system. Complete genomes were obtained. Strain E02-7-2 contained two plasmids (pE0272_KPC and pE0272_2), strain E01-7-1 contained one plasmid (pE0171_KPC), and strain E02-16-2 contained one plasmid (pE02162_KPC).

Plasmid pE0272_KPC, carrying *bla*_*KPC–*2_, is a 146,156-bp plasmid containing 191 predicted open reading frames (ORFs) and was assigned to IncQ1 (Supplementary Figure S3). It contains a 104-Kb backbone that shares 90% identity with 68 plasmid sequences in the NCBI database with a query coverage of greater than 50% (Supplementary Table S9). Of these 68 plasmids, 59 were from *K. pneumoniae*, three from *E. coli*, and seven from other species (Supplementary Table S9). Hence, the pE0272_KPC backbone is mainly distributed in and possibly derived from *K. pneumoniae*. The multidrug resistance (MDR) region of plasmid pE0272_KPC was composed of mobile elements flanked by two IS*26* sequences and contained a diversity of antibiotic resistance genes. The *bla*_*KPC–*2_ was located on an IS*26*-based composite transposon in pE0272_KPC, which was generated from a Tn1722-based unit transposon flanked by the insertion of two directly repeated IS26 elements, resulting in truncation of resolvase of Tn3 and Tn1722.

The second plasmid in strain E02-7-2 is pE0272_2, a 113,709-bp plasmid with a G+C content of 54%, and is assigned to multi-replicons, including IncFIA and IncFIB. The backbone of pE0272_2 shared a 62.5-Kb backbone with 70 plasmids from the NCBI database (Supplementary Table S9). Of these 70 plasmids, 60 were found in *E. coli*, five in *K. pneumoniae*, and the rest in other species (Supplementary Table S9). Therefore, pE0272_2 is likely to be of *E. coli* origin, in contrast to pE0272_KPC. Plasmid pE0272_2 does not contain *bla*_*KPC–*2_.

**FIGURE 3:**
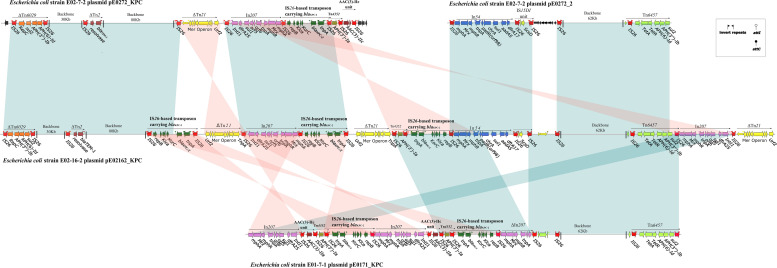
Linear comparison of sequenced plasmids. Genes are denoted by arrows and are colored based on the gene function classification. Shaded regions denote regions of homology (>99% nucleotide similarity). The backbone sequence is denoted by a shortened black line, not to scale.

Plasmid pE02162_KPC in strain E02-16-2 is a 275,546-bp plasmid containing 347 predicted ORFs (Supplementary Figure S4) and is assigned to multi-replicons, including IncFIA, IncFIB, and IncQ1. Plasmid pE02162_KPC is a composite of plasmids pE0272_KPC and pE0272_2 (>98% query coverage, >99% identity) and has two sets of genes involved in replication, plasmid partition, and conjugative transfer. The MDR regions of pE02162_KPC share 99% identity with pE0272_KPC and pE0272_2, both of which are composed of IS26-mediated transposons, although they differ in arrangements and the copy number of mobile elements (Figure 3). Each MDR region of pE02162_KPC is joined to the backbones of pE0272_KPC and pE0272_2 in a head-to-tail fashion (Figure 3). The relationship between the plasmid backbone and the MDR regions indicate that pE0272_KPC and pE0272_2 might be integrated into strain E02-16-2 as pE02162_KPC in the MDR regions, mediated by IS*26* (Figure 3 and Supplementary Figure S5). The *bla*_*KPC–*2_ in pE02162_KPC was located on the same IS26-based composite transposon as pE0272_KPC, with three copies.

Strain E01-7-1 contained pE0171_KPC, a 133,709-bp plasmid assigned to multi-replicons, including IncFIA and IncFIB (Supplementary Figure S6). The pE0171_KPC backbone shared 99% sequence identity with pE0272_2 but shared no identity with pE0272_KPC (Figure 3 and Supplementary Figure S5). Therefore, pE0171_KPC is also likely to be of *E. coli* origin. The MDR region of pE0171_KPC has identical IS*26*-mediated transposons as pE0272_KPC and pE02162_KPC, indicating that pE0171_KPC acquired antibiotic resistance genes, including *bla*_*KPC–*2_, via mediation by IS26. The MDR region of pE0171_KPC might have originated from the resolution of the plasmid cointegrate or the transmission of IS26-mediated transposons.

Illumina reads mapping combined with hybridization results showed that five strains contained the backbone of pE0171_KPC and 37 strains contained the backbone of pE0272_KPC and pE0171_KPC in the remaining 42 strains (Figure 2). Since most of the strains contained the pE0272_KPC sequence, and since the plasmid is mainly distributed in *K. pneumoniae*, it is possible that *E. coli* acquired this plasmid from *K. pneumoniae*. Seven strains with pE0171_KPC as *bla*_*KPC–*2_-positive plasmid were isolated after September 15, 2012 (Figure 2), suggesting that plasmid pE0272_2 acquired *bla*_*KPC–*2_ from pE0272_KPC, producing plasmid pE0171_KPC, and then disseminated. We further demonstrated that two *bla*_*KPC–*2_-carrying plasmids, pE0272_KPC and pE0171_KPC, were both successfully transferred to *E. coli* EC600. The transconjugants exhibited significantly reduced carbapenem susceptibility (Supplementary Table S10).

## Discussion

In this study, we examined an outbreak of infections caused by a carbapenem-resistant *E. coli* ST131 clone that persisted for 18 months in a hospital in Beijing. *E. coli* ST131 caused predominantly (76%) respiratory infections; the remainder of infections (24%) were UTIs or bacteremia. ST131 is a well-known clone causing UTIs but has been less frequently reported in causing respiratory and other infections. Our study is the first report of a nosocomial outbreak of ST131 carrying *bla*_*KPC–*2_. *E. coli*, especially ST131, has been shown to spread between and within hospitals and communities (Nicolas-Chanoine et al., 2014). Furthermore, ST131 can be transmitted via direct host-to-host contact and even via domestic pets (Johnson et al., 2009; Hilty et al., 2012). Our study demonstrates multiple challenges in controlling MDR ST131 infections in hospitals and communities. In 2013, we communicated our preliminary results to the hospital, which in turn adopted stricter infection control measures. The transmission of ST131 containing *bla*_*KPC–*2_ was controlled, and no *E. coli* carrying *bla*_*KPC–*2_ was detected in sputum samples during the following year.

The *E. coli* ST131 subclone H30 (*fimH*-based putative clonal lineages) is a major UTI pathogen that has been associated with multiple drug-resistance profiles (Tchesnokova et al., 2019). The outbreak we report here was also due to subclone H30, and our strains also presented with an MDR phenotype, highlighting the difficulty of treating respiratory tract infections, clinical failure due to inappropriate antibiotic treatment, and subsequent persistence of the clone within the hospital.

The *E. coli* ST131 strains described here were initially prevalent only in ward E02. Six SNPs separated the two clusters. Cluster II replaced Cluster I and became dominant in ward E02. Using whole-genome sequencing, we infer that the transfer of the patient carrying *E. coli* with *bla*_*KPC–*2_ triggered the spread of the ST131 clone, causing an outbreak in multiple wards.

To our knowledge, this is the first time the spread of *bla*_*KPC–*2_ has been fully described by genetically characterizing plasmid sequences from a carbapenem-resistant *E. coli* outbreak. There were three types of *bla*_*KPC–*2_-carrying plasmids found in *E. coli* ST131 in this outbreak: *K. pneumoniae*-derived plasmid pE0272_KPC, *E. coli*-derived plasmid pE0171_KPC, and a composite plasmid pE02162_KPC. Based on the distribution of pE0272_KPC-like plasmids, pE0272_KPC and, by association, the *bla*_*KPC–*2_ gene were presumably derived from *K. pneumoniae*. The *E. coli* plasmid pE0171_KPC backbone sequence has been frequently found in pathogenic *E. coli* (Ho et al., 2015; Li et al., 2015). The pE0171_KPC plasmid could acquire *bla*_*KPC–*2_ from a *K. pneumoniae*-derived plasmid containing the same MDR region but different backbone as pE0272_KPC. Furthermore, these plasmids have been shown to be self-transmissible, with an intact transfer region, suggesting that these plasmids maintain the ability to transfer between different strains carrying *bla*_*KPC–*2_.

The third *bla*_*KPC–*2_-containing plasmid pE02162_KPC is likely to be cointegrated by *E. coli*-derived and *K. pneumoniae*-derived plasmids during the hospital outbreak, as only one of the strains carried the plasmid. Plasmid cointegrate formation is a type of plasmid evolution involved in the development of antimicrobial resistance, virulence, and other traits (Wang et al., 2013). Plasmid p17-15-vir previously reported was a super hybrid plasmid with multiple replicons and carrying antimicrobial resistance genes, virulence determinants and conjugational systems, resulting in enhanced ability of transfer and risk (Li et al., 2020). IS26 appears to play a vital role in formation and resolution of plasmid cointegrate (He et al., 2015). Our results indicated that the pE02162_KPC plasmid is the product of IS26-mediated cointegration of pE0272_2 and pE0272_KPC. The cointegrate could subsequently resolve, with carbapenem resistance gene assigned to pE0272_2, resulting in pE0171_KPC. An *E. coli*-derived plasmid would be more suitable for the spread of *bla*_*KPC–*2_ in *E. coli.*

In conclusion, *E. coli* ST131 with *bla*_*KPC–*2_ was a multi-drug resistant epidemic clone during the nosocomial infection outbreak that can cause hospital-acquired pneumonia infections. Acquisition of multiple transmissible *bla*_*KPC–*2_-carrying plasmids resulted in a carbapenem-resistant ST131 clone that could rapidly disseminate in hospitals. Our results indicated that close monitoring of *bla*_*KPC–*2_-positive ST131 in hospitals is necessary for the control and proper treatment of ST131 infections.

## Data Availability Statement

The datasets presented in this study can be found in online repositories. The names of the repository, repositories, and accession number(s) can be found in the article/Supplementary Material.

## Author Contributions

JLi and JF conceived the study and designed experimental procedures. LG, MY, XC, XZ, JC, XB, and YZ performed the experiments. NT, WZ, and RL analyzed the data. DC, HZ, and HX contributed reagents and materials. JF, KS, NT, JLi, RL, TW, JLu, and JX wrote the manuscript. All authors contributed to the article and approved the submitted version.

## Conflict of Interest

The authors declare that the research was conducted in the absence of any commercial or financial relationships that could be construed as a potential conflict of interest.

## Footnotes

1 https://clsi.org/

2 https://pubmlst.org/

3 https://inkscape.org
