# Supplementary figures and images for: A Nosocomial Respiratory Infection Outbreak of Carbapenem-Resistant Escherichia coli ST131 With Multiple Transmissible blaKPC–2 Carrying Plasmids
Source: Front Microbiol. 2020 Sep 11;11:2068. doi: 10.3389/fmicb.2020.02068 (PMC7516988; doi:10.3389/fmicb.2020.02068)

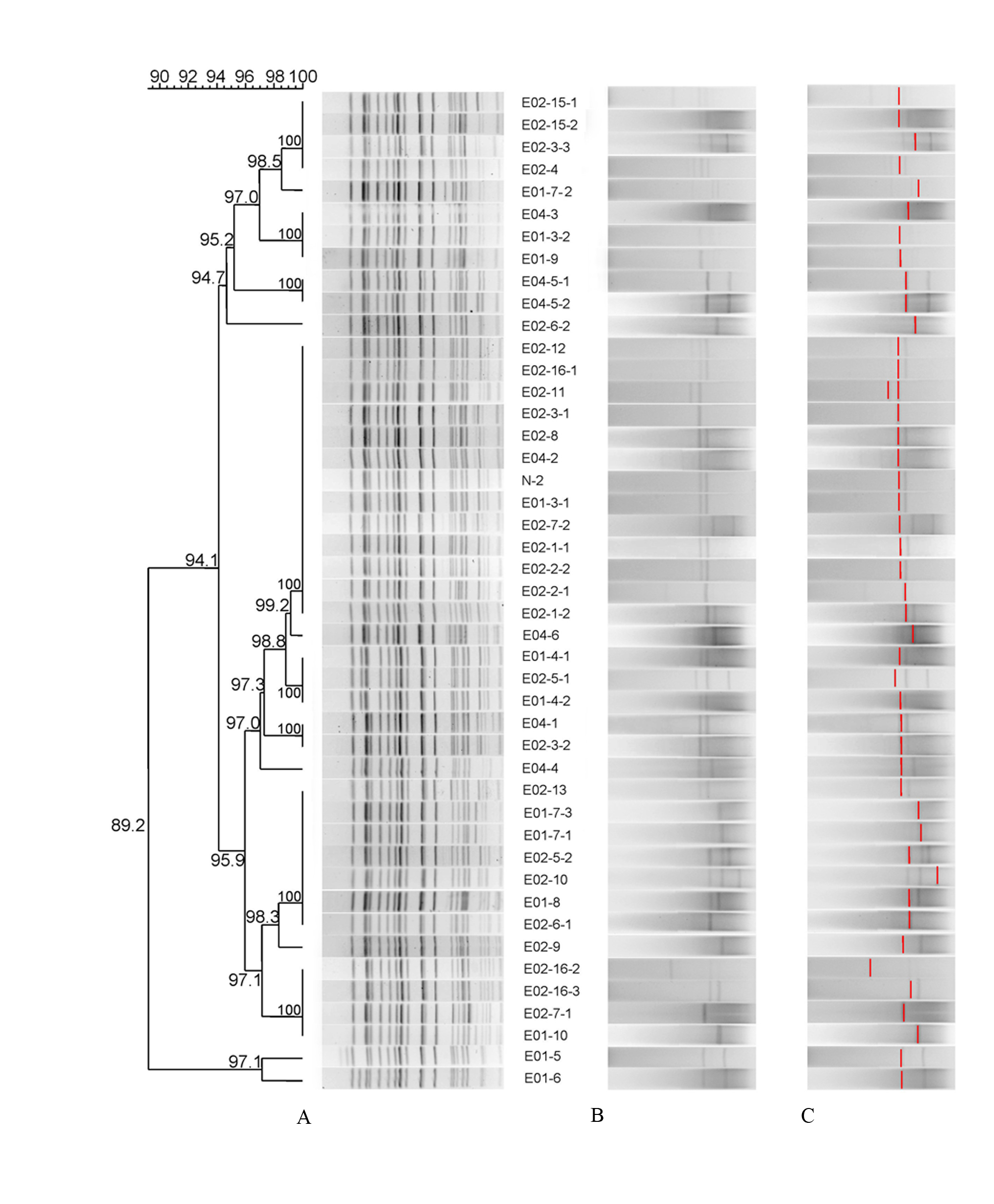

Supplement: Supplementary file 1 [file Image_1.TIF]

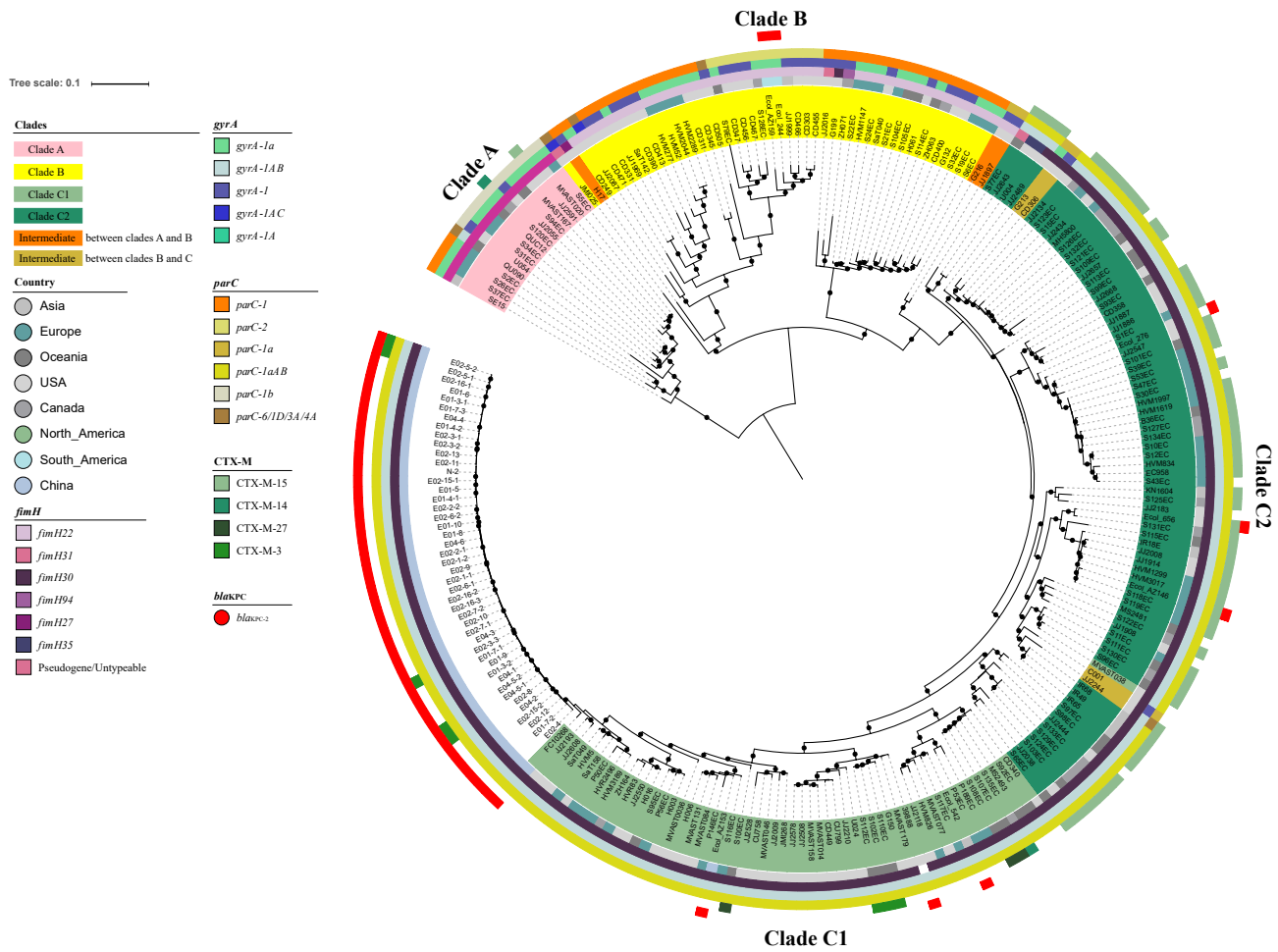

Supplement: Supplementary file 2 [file Image_2.pdf]

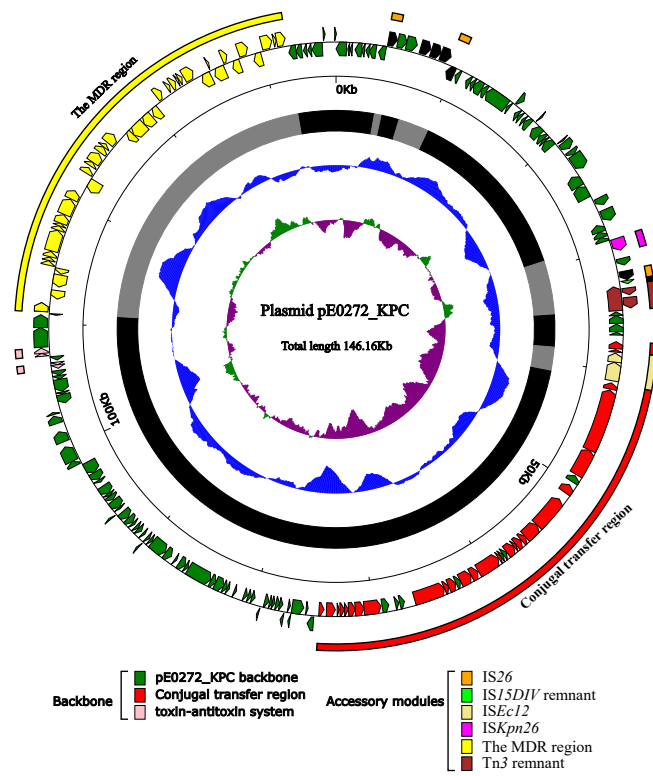

Supplement: Supplementary file 3 [file Image_3.pdf]

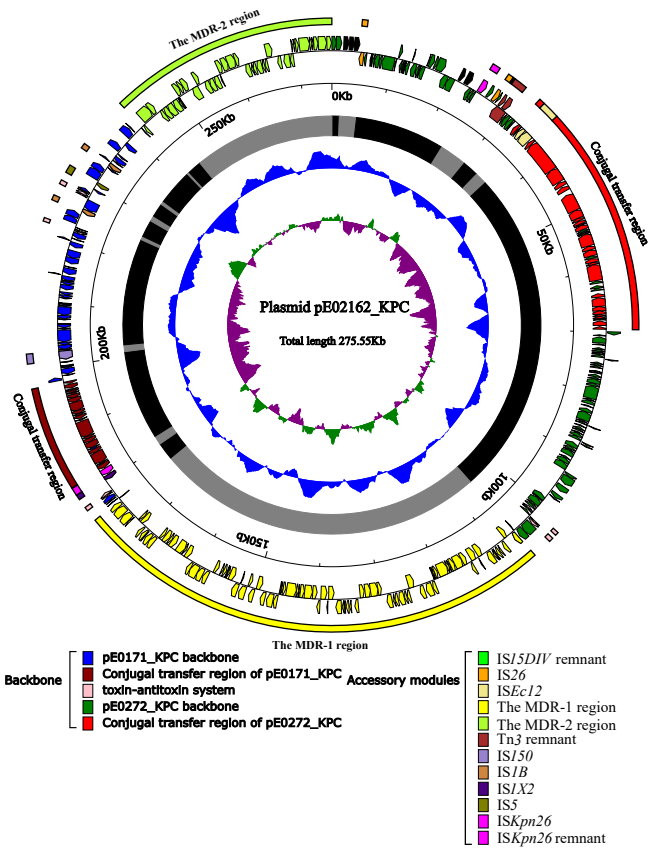

Supplement: Supplementary file 4 [file Image_4.pdf]

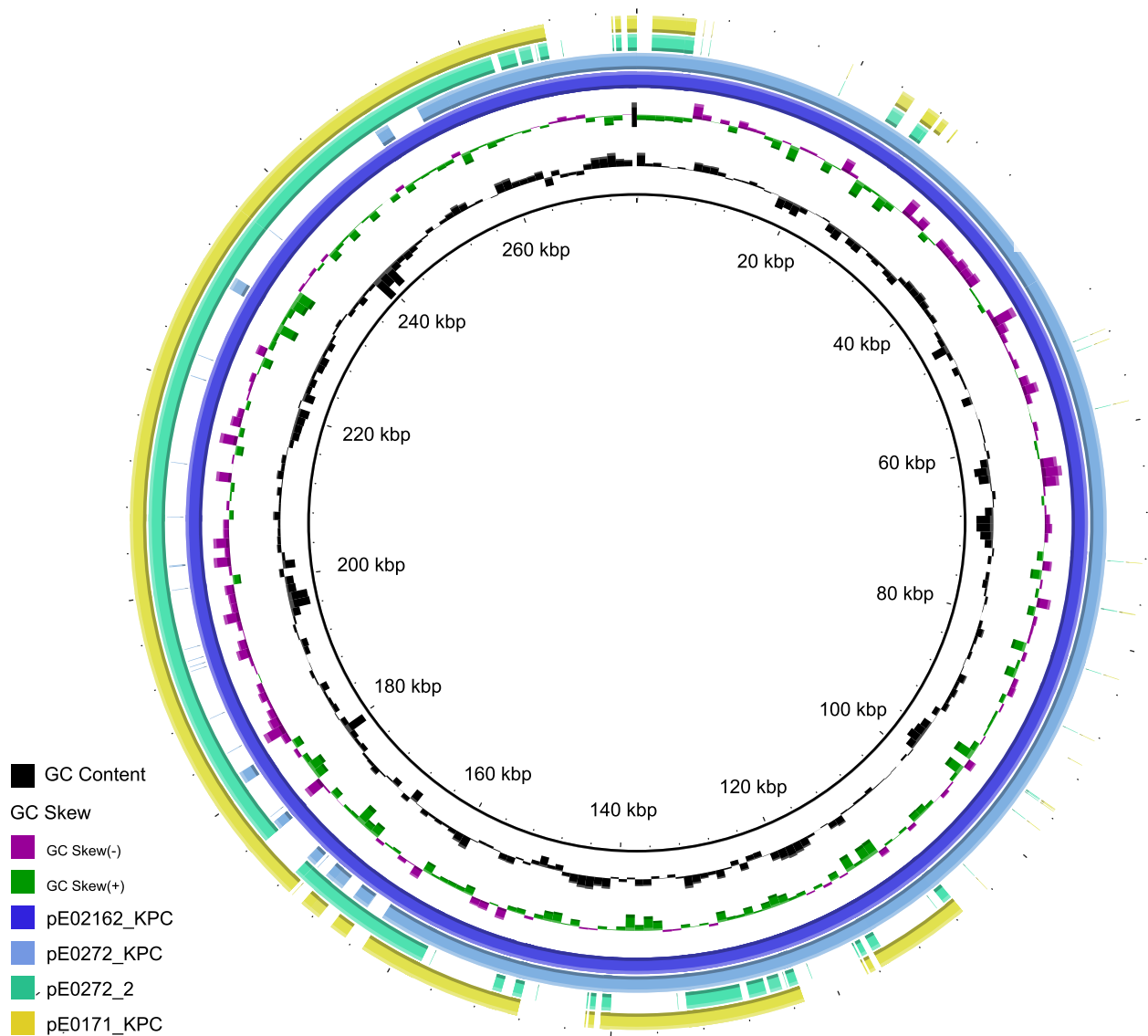

Supplement: Supplementary file 5 [file Image_5.pdf]

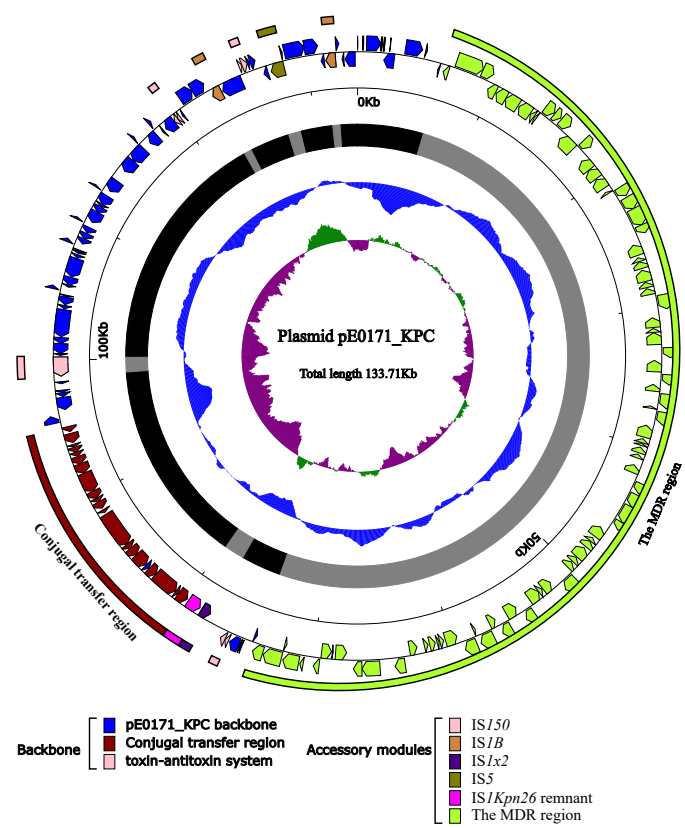

Supplement: Supplementary file 6 [file Image_6.pdf]
