# Supplementary material for: A Nosocomial Respiratory Infection Outbreak of Carbapenem-Resistant Escherichia coli ST131 With Multiple Transmissible blaKPC–2 Carrying Plasmids
Source: Front Microbiol. 2020 Sep 11;11:2068. doi: 10.3389/fmicb.2020.02068 (PMC7516988; doi:10.3389/fmicb.2020.02068)
Supplement: Supplementary file 7 [file Table_1.DOCX]

**Figure S1. The XbaI-PFGE, S1-PFGE electrophoresis patterns, and sketch map of S1-PFGE southern blot hybridization with the probe of *bla*_KPC-2_ gene of the 45 ST131 *E. coli* strains**

A. XbaI-PFGE electrophoresis patterns of 45 carbapenem-non-susceptible ST131 *E. coli* strains.

B. S1-PFGE electrophoresis patterns showing plasmid profiles of the 45 carbapenem-non-susceptible ST131 *E. coli* strains

C. Sketch of S1-PFGE southern blot hybridization results with the probe of *bla*_KPC-2_ gene. Positive band was shown in red.

**Figure S2. The phylogenetic tree of 242 ST131 *E. coli* strains.** The nodes highlight in color are 197 ST131 strains from NCBI database. The nodes without highlight are 45 carbapenem-non-susceptible *E. coli* strains in this study. Allelic profiling information is shown as colored strips surrounding the phylogram (from inner to outer) for the country, *fimH*, *parC*, *gyrA*, CTX-M, and *bla*_KPC-2_ genes. Two additional distinctions were made for some *fimH* variants: “Untypeable” corresponds to a strain with a truncated or missing *fimH* gene, and “Pseudogene” corresponds to a strain in which *fimH* is disrupted by an insertion sequence.

**Figure S3. The circular diagram of plasmid p0272_KPC sequence.** ORFs are shown as arrows. Blue, GC skew. Purple, GC content.

**Figure S4. The circular diagram of plasmid pE02162_KPC sequence.** ORFs are shown as arrows. Blue, GC skew. Purple, GC content.

**Figure S5. Sequence comparison of plasmid pE02162_KPC with pE0272_KPC, pE0272_2 and pE0171_KPC.**

**Figure S6. The circular diagram of plasmid pE0171_KPC sequence.** ORFs are shown as arrows. Blue, GC skew. Purple, GC content.
